# The Impact of the COVID-19 Pandemic on Myopia Progression in Children: A Systematic Review

**DOI:** 10.7759/cureus.28444

**Published:** 2022-08-26

**Authors:** Adrienne R Cyril Kurupp, Anjumol Raju, Gaurav Luthra, Mahrukh Shahbaz, Halah Almatooq, Paul Foucambert, Faith D Esbrand, Sana Zafar, Venkatesh Panthangi, Safeera Khan

**Affiliations:** 1 Pediatrics, California Institute of Behavioral Neurosciences & Psychology, Fairfield, USA; 2 Internal Medicine, California Institute of Behavioral Neurosciences & Psychology, Fairfield, USA; 3 Dermatology, California Institute of Behavioral Neurosciences & Psychology, Fairfield, USA

**Keywords:** refractive error, myopia, children, covid-19, pandemic

## Abstract

Myopia is the most common refractive error among children. The coronavirus disease 2019 (COVID-19) pandemic has affected children's health in many ways. Policy changes due to the COVID-19 pandemic, such as home quarantine and online schooling, have been proposed as causes for the increased risk of myopia progression. During strict home quarantine, children spend less time outdoors and more time using electronic devices which are important risk factors associated with myopia. Our systematic review aims to assess the relationship between myopia progression and these risk factors in children.

We did the literature search from PubMed, Google Scholar, and ScienceDirect. A total of 10 research papers were selected for final review using the Preferred Reporting Item for Systematic Review and Meta-Analyses (PRISMA) guidelines. The research articles used had a quality of more than 70%. The quality of these articles was determined using the Joanna Briggs Institute (JBI) tool. Our review included eight cross-sectional and two cohort studies. Most of these studies used questionnaires to assess the risk factors of myopia. Standardized ocular examinations were done in most studies to measure visual acuity, spherical equivalent, and axial lengths. Our study found that the progression of myopia was affected by the reduced time spent outdoors and increased screen time during the pandemic. We also found that children's increased use of electronic devices, such as mobile phones and tablets, has significantly affected myopia progression during the pandemic.

## Introduction and background

Myopia is a rising health concern affecting children in the modern world. Around 80 to 90 percent of children in East Asian countries and one-third of adults in the United States of America (USA) and Europe are myopic [1]. The prevalence of myopia has increased significantly during the past decade, with a higher predominance in Asian than in Western countries [2]. If the current trend continues, the World Health Organization (WHO) predicts that 50 percent of the world may be myopic by 2050 [3-5]. 

Studies have shown that the complex interplay of genetic, environmental, and behavioral factors results in the onset and progression of myopia [6]. Evidence suggests that increased screen time, sustained near work, and decreased time spent outdoors is associated with myopia development [3,7]. Other risk factors include age, gender, history of myopia in parents, and psycho-social stress [1]. 

Myopia was considered a benign condition previously. Recent research has shown that some cases of myopia can even lead to pathological complications such as cataracts, glaucoma, retinal detachment, and maculopathy [2,8]. According to WHO, the second largest cause of vision impairment and vision loss is an uncorrected refractive error [5]. The complications associated with myopia can severely impact the quality of life and may increase the economic burden of the individual. In the USA, the approximate financial burden of eye diseases is around $139 billion [8]. Therefore, it is necessary to study more about myopia and its risk factors. This way, we can avoid ocular complications and reduce the global economic burden.

The recent coronavirus disease 2019 (COVID-19), also known as the Severe Acute Respiratory Syndrome Coronavirus-2 (SARS-CoV-2) pandemic, has affected people's lives in many ways. Governments all around the globe implemented strict home quarantine measures to control COVID-19 infection. Much research has been conducted to study the effects of COVID-19 disease and quarantine measures on people's physical and mental well-being.

Even though the pandemic did not directly affect myopia, the associated home quarantine and online schooling notably affected myopia progression. Myopia which developed during the COVID-19 home quarantine is sometimes called 'Quarantine myopia' and has increased in prevalence and severity [3]. The reduced time spent outdoors, increased near work, increased time spent on electronic gadgets, and online learning are some of the contributing factors [3-4,9]. Recent studies have found a significant association between myopic progression and policies during the pandemic by governments [10]. 

Our systematic review aims to study the effects of the pandemic on myopia in children and its progression. We explored various risk factors associated with myopia progression, especially the impact of screen time and time spent outdoors. We also aimed to study if the type of electronic gadgets used by children is associated with myopia progression.

## Review

Methods

This study followed the recommended guidelines of Preferred Reporting Item for Systematic Review and Meta-Analyses (PRISMA) [11]. The inclusion and exclusion criteria, the databases, and keywords for the literature review were discussed with all the authors.

Inclusion and Exclusion Criteria

Inclusion: Pediatric population; Topics related to the study; Research articles published from 2019 to 2022; Articles published before the start date of April 10, 2022

Exclusion: Adult population; Reviews, meta-analysis, case reports, case series, letters to the editor, and commentary; Animal studies

Search Strategy

We conducted a systematic literature search in PubMed, Google Scholar, and ScienceDirect. We used Medical Subject Headings (MeSH) keywords to search PubMed and advanced search strategies in Google Scholar and ScienceDirect. Our search yielded sufficient relevant articles on the topic.

The following search terms were used in each database: (1) PubMed: pandemic, COVID-19, SARS-CoV-2, refractive errors, and myopia; (2) Google Scholar: pandemic, COVID-19, refractive errors, myopia, and children; (3) ScienceDirect: pandemic, COVID-19, SARS-CoV-2, refractive errors, and myopia.

Results

Data Extraction 

We extracted 223,567 articles from the three databases using the search strategies - 203,186 from ScienceDirect, 19,767 from PubMed, and 614 from Google Scholar. After applying filters in all three databases and removing duplicates, we selected 1,337 articles. At this stage, two authors - ARCK and AR - reviewed these articles independently and selected 69 relevant research articles. The screening was done by reading the titles and abstracts of the research articles. From these 69 articles, we chose 18 research articles for the quality check after applying inclusion and exclusion criteria and full-text availability. A consensus was achieved among the two authors while selecting the articles. 

Quality Assessment

**Figure 1:**
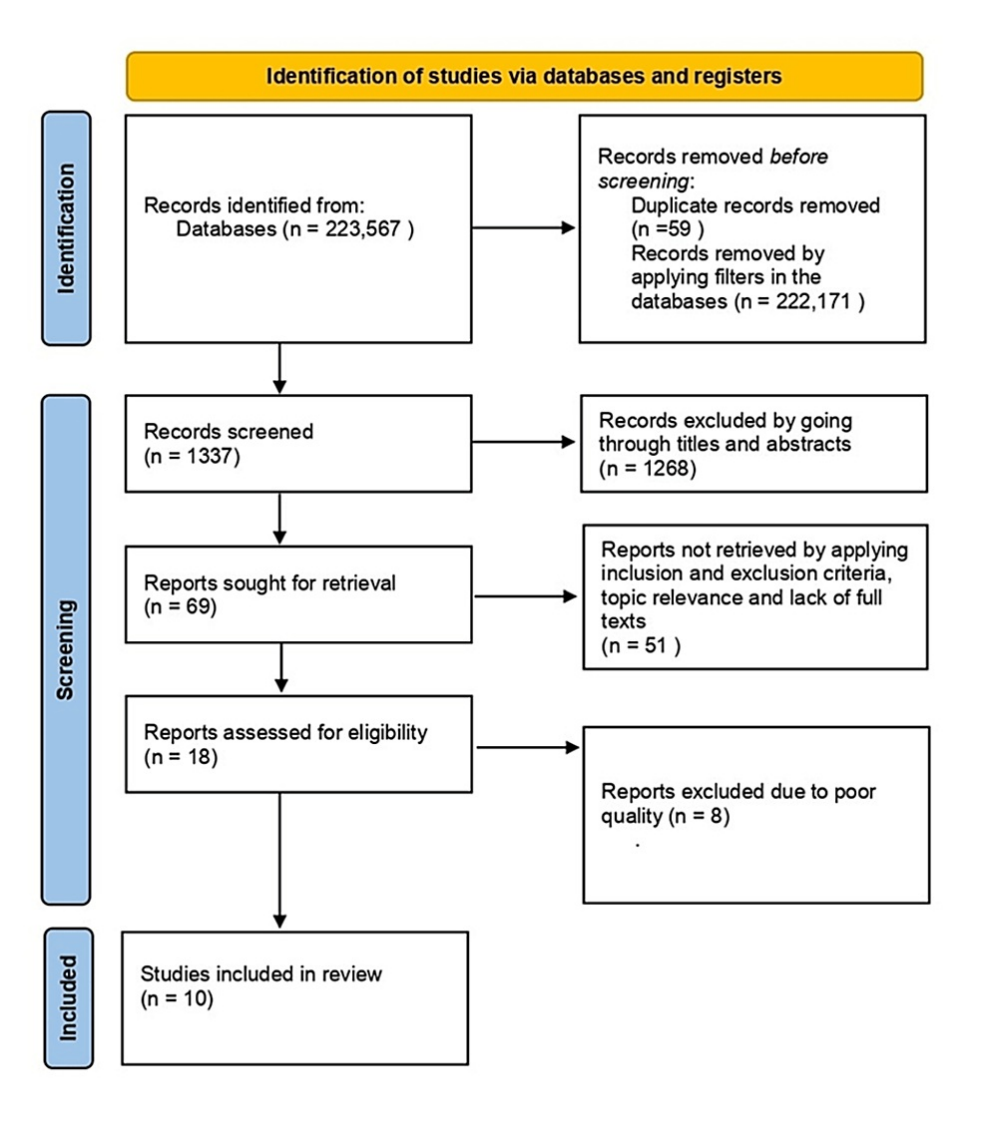
The selection process diagram using PRISMA guidelines PRISMA - Preferred Reporting Item for Systematic Review and Meta-Analyses n - number of articles

The 18 articles selected included 15 cross-sectional and three cohort studies. The Joanna Briggs Institute's (JBI) quality appraisal questionnaires were used to test the quality of these articles. We finalized 10 research articles for our systematic review with more than 70% quality. Figure 1 explains the selection process of these 10 papers.

Characteristics and Outcomes of the Included Studies

Ten studies selected for the final review included eight cross-sectional and two cohort studies. Six of these studies were conducted in China [1,12-16]. The other studies were conducted in India [17], Tibet [18], Hong Kong [19], and Turkey [3]. The number of participants ranged from 115 to 1,001,749. Age groups studied included primary school children, high school students, and university students. We included the studies published between 2019 to 2022. Most studies used questionnaires to study the risk factors associated with myopia progression. They also used standard ophthalmologic tests to measure axial length, spherical equivalent, and visual acuity. Standardized charts were used to test visual acuity. Cycloplegic and non-cycloplegic techniques were used to measure refraction in these studies. One study used only self-reported questionnaires to assess myopic symptoms and exposure to risk factors [1]. A few studies used values from only the right eyes for statistical analysis [12-14,16,18]. One study used results from the more myopic eye of the study subjects [15]. Most studies recorded and compared values before and during the COVID-19 home quarantine and this made it easier to assess the risk factors of myopia progression between the two timeframes. Table 1 shows the characteristics and results of the 10 research articles in our systematic review.

**Table 1:** The characteristics and results of research articles used in this systematic review COVID-19: coronavirus disease 2019; SE: spherical equivalent; D: diopter; UCVA: uncorrected visual Acuity; N/A: not applicable, h/day: hours/day; WHO: World Health Organization

Author	Study title	Study type	Country of study	Age of the study population	Sample size	Study results
Wang W et al. [12]	Survey on the progression of myopia in children and adolescents in Chongqing during COVID-19	Cross-sectional	China	Students from grades one to six in primary school, grades one to two in junior school, and grades one to two in high school	1,728 in 2019, 1,733 in 2020	The percentage of myopia in 2020 was 10.40% higher than in 2019. The SE in 2020 (−1.94 ± 2.13 D) after the home quarantine was higher than in 2019 (−1.64 ± 5.49 D). Students using computers and mobile phones for digital learning during the pandemic had worse UCVA and SE than children using other digital devices.
Cai T et al. [13]	A complex interplay between COVID-19 lockdown and myopic progression	Cross-sectional	China	N/A	115	Refractive error increased from 0.20 D to 0.45 D during the three-month home quarantine. A 35% faster progression was seen in the monthly axial length growth rate during the pandemic. Myopia progressed by ⅓ times or 33.33%, which can be attributed to increased screen time and decreased outdoor activities during home confinement.
Mohan A et al. [17]	The impact of online classes and home confinement on myopia progression in children during COVID-19 pandemic: digital eye strain among kids(DESK) study 4	Cross-sectional	India	6-18 years	133	Mean SE during the COVID-19 pandemic was -5.12 +/- 2.70 D and -4.54 +/- 2.70 D before the pandemic. History of rapid myopia progression, <1 h/day of sun exposure, and >1 h/day of video games and mobile phones were identified as possible risk factors for myopia progression during the COVID-19 pandemic.
Liu J et al. [1]	Examining risk factors related to digital learning and social isolation: youth visual acuity in COVID-19 pandemic	Cross-sectional	China	Primary, secondary, and university in China	3918	The average digital use during the pandemic was 3.91 h/day (which is more than the WHO recommendation). Myopia symptoms increased with every one-hour increase in digital device use.
Yao Y et al. [18]	Distribution, progression, and associated factors of refractive status of children in Lhasa, Tibet, after COVID-19 quarantine	Cross-sectional	Tibet	7.9 +/- 0.5 years	1819	When compared to pre-COVID times, the proportion of myopia progressed by 7.0% among the children in Tibet.
Xu L et al. [14]	COVID-19 quarantine reveals that behavioral changes affect myopia progression	Cross-sectional	China	7-18 years	1001749	Six-month myopia progression among the study subjects increased around 1.5 times from pre-COVID-19 times. Myopia progression was positively associated with increased screen time and negatively associated with outdoor activity.
Ma M et al. [15]	COVID-19 home quarantine accelerated the progression of myopia in children aged 7 to 12 years in China	Cross-sectional	China	7-12 years	201	The increase in myopia progression during the COVID-19 quarantine was three times higher than the baseline myopia progression. Myopia progression was associated with increased screen use during the home quarantine.
Aslan F et al. [3]	The effect of home education on myopia progression in children during the COVID-19 pandemic	Cross-sectional	Turkey	8-17 years	115	Annual progression analysis revealed a higher myopic progression in 2020 (after the COVID-19 quarantine) than in 2019 and 2018 (before COVID-19).
Ma D et al. [16]	Progression of myopia in a natural cohort of Chinese children during the COVID-19 pandemic	Cohort	China	8-10 years	Study group - 208 Control group-83	The mean myopia progression before the pandemic was -0.3 D which increased to -0.9D during the pandemic.
Zhang X et al. [19]	Myopia incidence and lifestyle changes among school children during the COVID-19 pandemic: a population-based prospective study	Cohort	Hong-Kong	6-8 years	Study group - 709 Control group - 1084	In the pre-COVID-19 cohort, the estimated annual incidence of myopia was 11.63%, which is less than the COVID-19 cohort (29.68% ).

Discussion

The most common refractive error detected in children is myopia. It occurs due to the abnormal elongation of the eyeball. In the myopic eye, the refractive image of an object falls in front of the retina and appears blurry. There are two types of myopia - (i) pathological myopia and (ii) school-age myopia. School-age myopia is the most common type and progresses slowly. Pathological myopia is rare and can worsen rapidly to high refractive errors, typically more than -6.00 diopter (D) [20].

Genetic and environmental factors influence the development of myopia [2,8,13]. Its prevalence is also different in ethnic and geographic populations [2]. Myopia progression is also affected by seasonal changes [8,15]. During winter, as children spend more time indoors and less time outdoors, myopia progresses more [8,15]. Increased outdoor time and natural light exposure are protective factors preventing myopia progression [2]. Children with a family history of myopia have a higher risk of developing myopia [2,8,12]. 

The prevalence of myopia is on the rise due to increased educational status, increased stress, increased time spent on electronic devices such as laptops, tablets, and mobile phones, and decreased time spent outdoors [1,8,20]. Myopia is more common among urban communities, students, and computer professionals as they are more prone to near-work activities such as reading and more screen time [8]. 

The onset of the COVID-19 pandemic has affected the behaviors of children drastically since there was a shift from in-person education to online schooling and decreased outdoor activities due to home quarantine. These changes are new to us and we have not experienced anything like this in over 100 years. This necessitates the importance of further exploring these behavioral changes in children during the pandemic and the effects they might have on their ocular health. Our systematic review studies whether such changes can negatively affect children's myopia and worsen its progression. 

Myopia Progression During the Pandemic

The behavioral changes during the pandemic, such as online schooling and home quarantine, are anticipated to adversely affect children's myopia [1].

Wang W et al. found that the prevalence of myopia increased by 10.49% during the pandemic [12]. Ma D et al. found a myopia progression of -0.9 D in seven months during the home quarantine, whereas the average myopia progression before COVID-19 was only -0.3D [16]. Even though the study detected a significant change in spherical equivalent (SE), they did not find any significant change in the axial length (AL) and uncorrected visual acuity (UCVA). This change in only SE was considered transient due to accommodative spasm [16]. Whereas, a study conducted by Cai T et al. detected a 35% increase in the axial length of myopic students during the COVID-19 home confinement. They also found a rapid worsening of SE and severe asthenopia (dry eyes) during the same period. Severe asthenopia which developed during this time due to increased screen exposure and near-work was speculated to be the causative factor for the rapid increase in axial length [13]. 

According to Mohan A et al., 62.4% of the children had myopia progression during the pandemic, and only 45.9% of the study population showed myopia progression before COVID-19 [17]. Xu L et al., Ma M et al., Zhang X et al., and Aslan F et al. also found a higher rate of myopic progression during COVID-19 than before [3,14,15,19].

Previous studies conducted in Lhasa, Tibet, found that children in Lhasa have mild hyperopia at a baseline compared to children in nearby provinces. Tibetan (Lhasa) children are exposed to longer daylight hours, which is a protective factor that prevents myopia development and progression. A recent study by Yao Y et al. in Tibet showed only a seven percent increase in myopia progression. This myopia progression rate was much lower compared to more than 10% progression rates in Hong Kong and nearby provinces of China. The prevalence of myopia among Tibetan children has always been lower compared to children in nearby regions. The myopia progression rate during COVID-19 has been comparable to previous years. This can be due to the minimal baseline behavioral changes in Tibetan children even during home quarantine. The region studied (Lhasa) was least affected by COVID-19, with less strict quarantine measures. Lhasa had only one month of strict home quarantine and two hours of online classes. The children spent adequate time outdoors. All these factors are protective against myopia progression. In other words, we can say that strict home quarantine measures, online education, increased screen time, and less time spent outdoors experienced during the pandemic have a significant effect on myopia progression in children [18].

Time Spent Outdoors and Myopia Progression

With the widespread use of electronic devices and increased urbanization, children spend less time outdoors. The increased competitiveness in the school curriculum is another factor that makes children spend more time indoors. The home quarantine measures implemented to slow the spread of COVID-19 have further worsened the situation. Previous studies have shown that the time spent outdoors protects against myopia development and progression [21,22]. The time spent on outdoor activities has reduced significantly during COVID -19 pandemic [15-17,19]. This has been found to increase the rate of myopia progression in children [12,14,17]. 

The monthly axial growth rate was found to negatively correlate with outdoor light exposure by Cai T et al. Outdoor light exposure reduces the myopia progression rate as the natural light increases the dopamine release, which is beneficial in decreasing axial elongation of the eyeball [13]. 

Unlike the studies mentioned above, Ma D et al. and Ma M et al. did not find any association between outdoor time and myopia progression, even though they found a significant reduction in outdoor time. This difference could be due to the small sample size and the low baseline outdoor time among the study population [15,16].

The study results from Aslan et al. further strengthen the hypothesis that time spent outdoors is protective against myopia progression. The study found a decreased rate of myopia progression in children who spent at least two hours daily outside. The myopia progression rate was also less in children who lived in independent houses compared to those living in apartment buildings, which provides opportunities for children to spend more time outdoors [3]. These studies point out that time spent outdoors and natural light exposure reduces the incidence and progression of myopia in children. 

Screen Time and its Association with Myopia Progression

Most studies conducted to assess behavioral changes in children and adults during the COVID-19 home quarantine show a drastic increase in screen time. According to United Nations Educational, Scientific and Cultural Organization (UNESCO), 1.1 billion children in over 140 countries had digital device exposure from online learning and home quarantine restrictions, during the COVID-19 pandemic [3].

Recently, screen time among children and adolescents has increased and the situation has worsened during the pandemic. On average, the digital device use was 3.91 hours/day (h/day) in the study conducted by Liu Ji et al., which exceeded the daily recommended digital screen time of two h/day by WHO for children aged between five to 17 years old [1]. This necessitates the importance of exploring the possible association between increased screen time and ocular health. 

Ma D et al. found that children spent around 2.43 +/- 2.19 h/day on the digital screen and an additional 1.94 +/- 1.05 h/day on online education during the COVID- 19 pandemic. Before the pandemic, screen time was only 1.42 +/- 1.77 h/day [16]. Xu L et al. and Ma M et al. also report a significant increase in screen time among students during home quarantine. These studies also report a significant association between screen time duration and myopia incidence and progression [14,15].

An interesting finding in recent studies is the significant increase in time spent on near-viewing devices such as smartphones and tablets, and a decrease in time spent watching television and computers [12,17,19]. It was found that 96.7% of students in the study conducted by Mohan A et al. and more than 50% of students studied by Wang W et al., used smartphones for online learning [12,17]. During the pandemic, when schools shifted to online learning, more students started using devices such as smartphones and tablets, as they are more affordable than other devices such as laptops. This trend was seen more in developing countries like India, where mobile phone usage increased significantly during the COVID-19 home quarantine. The percentage of students who used smartphones for one to two days has increased from 52.63% to 68.62%. A significant association was found between rapid myopia progression and mobile use of more than one h/day [17]. 

The study by Liu J et al. demonstrates a positive association between myopia and each additional hour of digital device use [1]. The SE of children using handheld devices was higher than those using devices such as television [12,15]. Myopia progression worsened with increased use of near electronic devices [1,12,15]. However, Aslan F et al. did not find an association between myopia progression and screen time duration or the device type [3]. Most of the studies in our systematic review point out a significant increase in screen time, especially with the popularity of handheld devices. The increased use of handheld devices has been shown to affect myopia incidence and progression during the COVID-19 pandemic. 

Study Limitations

Most studies in this systematic review used questionnaires to study various risk factors associated with myopia progression which might have caused recall bias. The studies were conducted in different countries, so children's baseline behaviors might not be the same. Our review has not studied if the difference in baseline behaviors can affect the study outcome. 

## Conclusions

Our systematic review aimed to study myopia progression's two most important risk factors - time spent outdoors and screen time during the pandemic. We found that children spend less time outdoors and sometimes no time at all due to strict home quarantine rules. Screen time increased significantly during this period, as children used electronic devices for recreation and online learning. Our review found that children used near-viewing devices such as smartphones and tablets more than other electronic devices such as televisions or personal computers. We discovered that strict home quarantine, reduced time spent outdoors, and increased screen time are associated with myopia progression in children during the pandemic. Increased use of mobile phones and tablets was also associated with myopia progression. Therefore, educating children and parents on these risk factors and ways to tackle them is crucial. Educating them on reducing screen time, taking frequent breaks while using electronic gadgets, using lubricant eye drops to reduce dry eyes, and being encouraged to spend more time outdoors will help prevent further myopia progression. 
